# Supplementary figures and images for: Transient Poly(ADP-Ribose) Triggers FUS Condensation Hysteresis via a Prion-Like Mechanism
Source: bioRxiv. 2025 Jul 5:2025.07.03.659157. Preprint. [Version 1] doi: 10.1101/2025.07.03.659157 (PMC12236490; doi:10.1101/2025.07.03.659157)

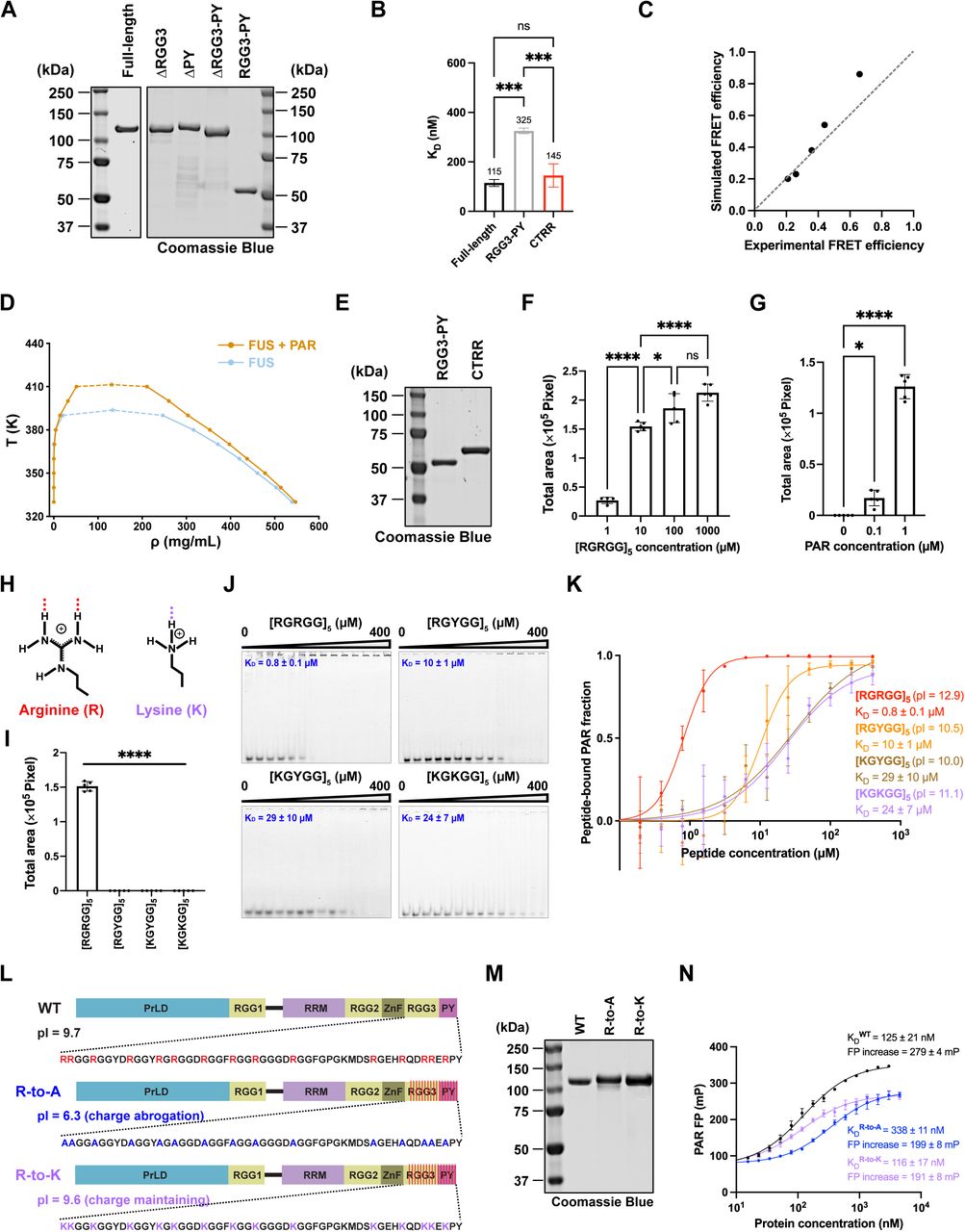

Supplement: Supplement 1 — Figure S1, related to Figure 1 (A) Representative SDS-PAGE (4% stacking gel and 10% resolving gel) with Coomassie Brilliant Blue staining showing the purified His-MBP-tagged full-length FUS and its mutants, as presented in Figure 1. The His-MBP solubility tag is specifically cleaved by tobacco etch virus (TEV) protease to initiate the condensation reaction. (B) Comparisons of KD values in Figure 1E. n = 3; error bars, SD; ordinary one-way ANOVA with Tukey's multiple comparisons test, not significant (ns) and p < 0.001 (***). (C) Correlation plot comparing FRET efficiency between simulations and experiments for different PAR lengths (see Methods). (D) Phase diagram of FUS condensation with and without PAR from slab simulations. The densities of the low-density phase (ρL, points on the left) and high-density phase (ρH, points on the right) are plotted against temperature. The critical temperature, where the two phases become indistinguishable (indicated by the top of each curve), is extrapolated from data points at various temperatures (see Methods). The critical temperature is approximately 394 K for FUS alone (blue curve) and approximately 411 K for the FUS + PAR system (amber curve). (E) Representative SDS-PAGE (4% stacking gel and 12% resolving gel) with Coomassie Brilliant Blue staining showing the purified His-MBP-tagged RGG3-PY fragment and the CTRR fragment, as presented in Figure 1. (F) Bar graph quantification of the total area covered by condensates formed in Figure 1H. n = 5; error bars represent SD; ordinary one-way ANOVA with Tukey's multiple comparisons test, with significance levels: not significant (ns), p < 0.05 (*), and p < 0.0001 (****). (G) Bar graph quantification of the total area covered by condensates formed in Figure 1I. n = 5; error bars represent SD; ordinary one-way ANOVA with Tukey's multiple comparisons test, with significance levels: p < 0.05 (*), and p < 0.0001 (****). (H) Schematic of arginine and lysine side chains showing charge and [file figure-S1.jpg]

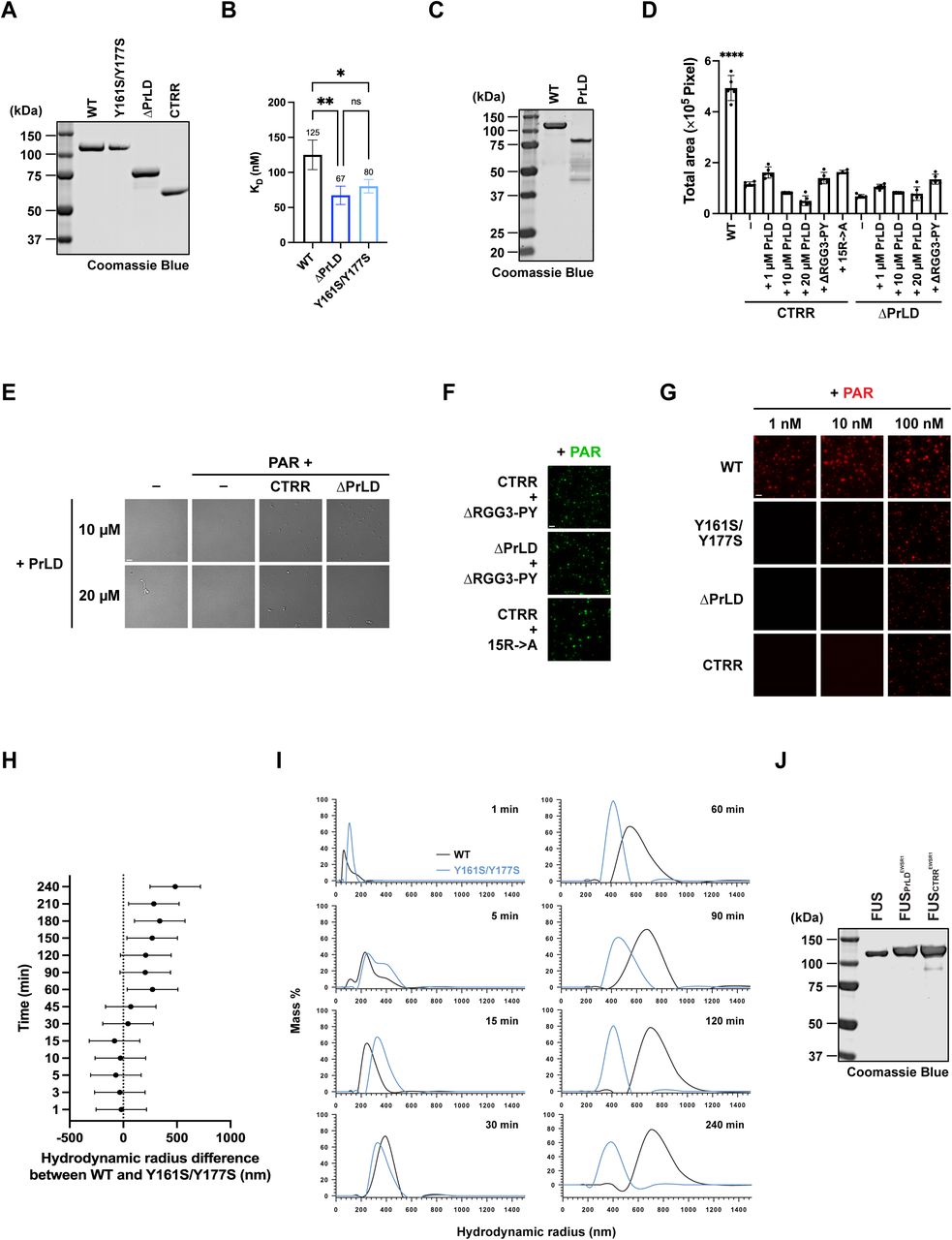

Supplement: Supplement 2 — Figure S2, related to Figure 2 (A) Representative SDS-PAGE (4% stacking gel and 10% resolving gel) with Coomassie Brilliant Blue staining showing the purified His-MBP-tagged full-length FUS and its mutants, as presented in Figure 2A. (B) Bar graph comparing KD values from Figure 2F. n = 3; error bars, SD; ordinary one-way ANOVA with Tukey's multiple comparisons test, with significance levels: not significant (ns), p < 0.01 (**), and p < 0.001 (***). (C) Representative SDS-PAGE (4% stacking gel and 12% resolving gel) with Coomassie Brilliant Blue staining showing the purified His-MBP-tagged full-length FUS and its PrLD. (D) Bar graph quantification of the total area covered by condensates formed in Figures 3G, S3E and S3F. n = 5; error bars represent SD; ordinary one-way ANOVA with Tukey's multiple comparisons test, p < 0.0001 (****) between wild-type FUS and all the other conditions. (E) Representative DIC microscopic images of condensates formed by adding 10 or 20 μM PrLD with the indicated additives. When included, CTRR and ΔPrLD are at 1 μM, and PAR is at 100 nM. n = 5. Scale bar, 5 μm. See also (D). (F) Representative fluorescence microscopic images showing condensation results for 1 μM of each protein adding in trans with 100 nM PAR. Fluorescence is from Cy3-labeled PAR. n = 5. Scale bar, 5 μm. See also (D). (G) Fluorescence microscopy images showing Cy3-PAR signal corresponding to Figure 2H. n = 5. Scale bar, 5 μm. (H) Forest plot showing the hydrodynamic radius differences between condensates formed by wild-type FUS and the Y161S/Y177S mutant with PAR at different time points, corresponding to the two-way ANOVA analysis in Figure 2J. n = 3; error bars represent Šidák-adjusted 95% confidence intervals (intervals including 0 indicate no significant difference). (I) Representative dynamic light scattering (DLS) mass distribution plots by hydrodynamic radius at the indicated time points, corresponding to Figure 2K. Wild-type FUS and the Y161S/Y177S mutant are com [file figure-S2.jpg]

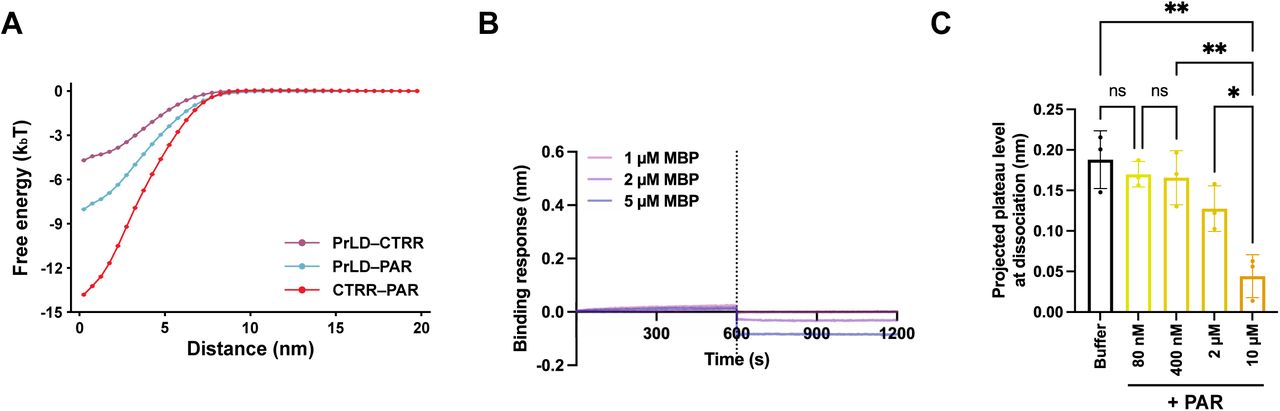

Supplement: Supplement 3 — Figure S3, related to Figure 3 (A) Free energy profiles for interactions between PrLD, CTRR, and PAR in umbrella sampling simulations, corresponding to the calculated binding free energies shown in the bar chart in Figure 3E (see Methods). n = 5; error bars, SD. (B) Representative BLI sensorgram showing no positive binding responses of PrLD and MBP. n = 3. (C) Comparison of the projected plateau levels at dissociation by the introduction of buffer and titration of PAR, corresponding to Figure 3H. n = 3; error bars, SD; ordinary one-way ANOVA with Tukey's multiple comparisons test, with significance levels comparing the projected plateau level at dissociation: not significant (ns), p < 0.05 (*) and p < 0.01 (**). [file figure-S3.jpg]

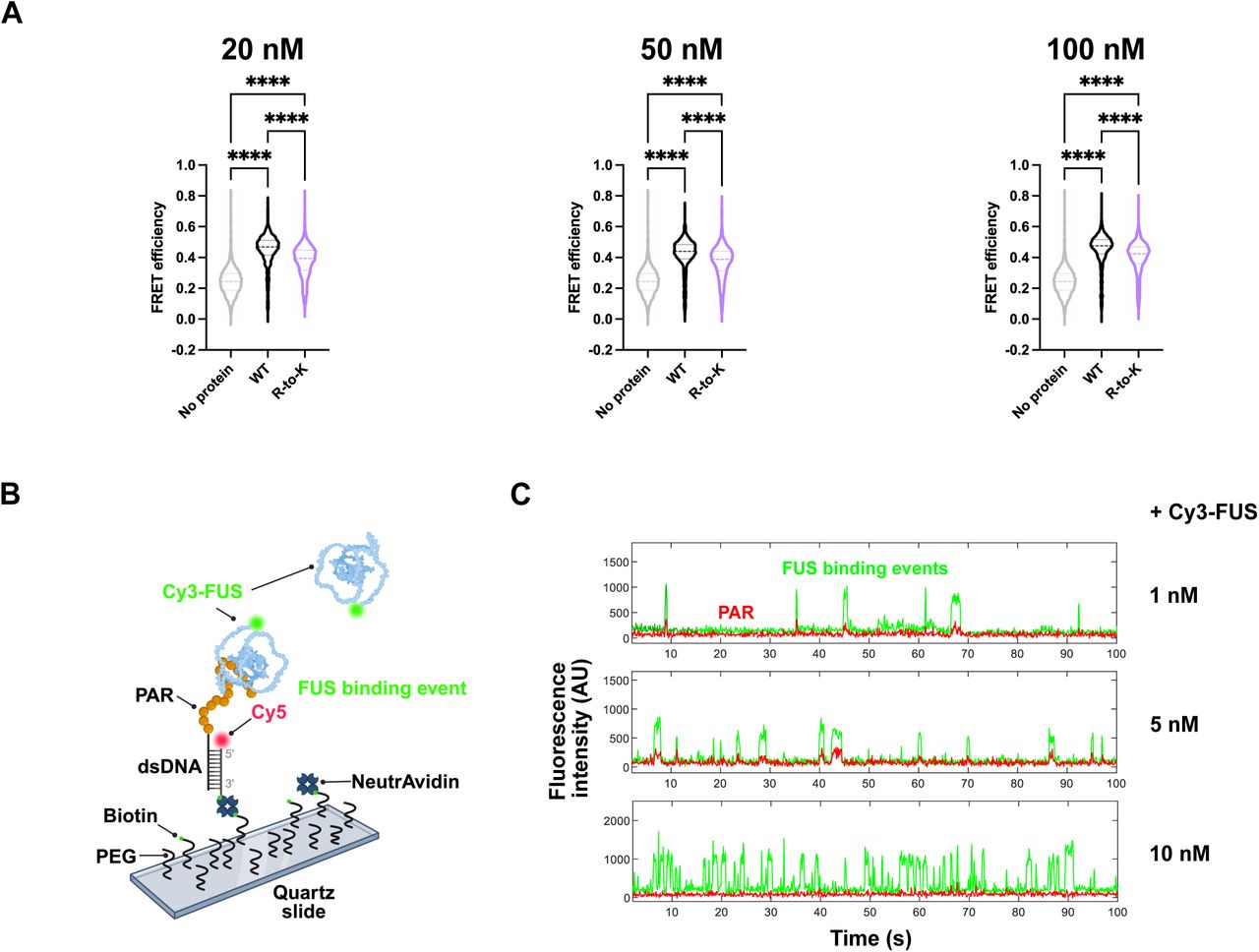

Supplement: Supplement 4 — Figure S4, related to Figure 4 (A) Violin plots of PAR FRET efficiencies in response to protein concentrations of 20 nM, 50 nM, and 100 nM. n > 800; dashed lines represent the 75th percentile, the median (50th percentile), and the 25th percentile; Kruskal-Wallis test, p < 0.0001 (****). (B) Schematic of single-molecule total internal reflection fluorescence (TIRF) microscopy measurement of Cy3-FUS binding to immobilized PAR, with Cy5-labeled double-stranded DNA (dsDNA) serving as a scaffold. FUS binding to PAR can be detected by the increase in Cy3 signal. (C) Representative single-molecule fluorescence traces of Cy3 (green, FUS) and Cy5 (red, immobilized PAR) at increasing Cy3-FUS concentrations. Cy3 signal bursts correspond to transient FUS binding events. [file figure-S4.jpg]

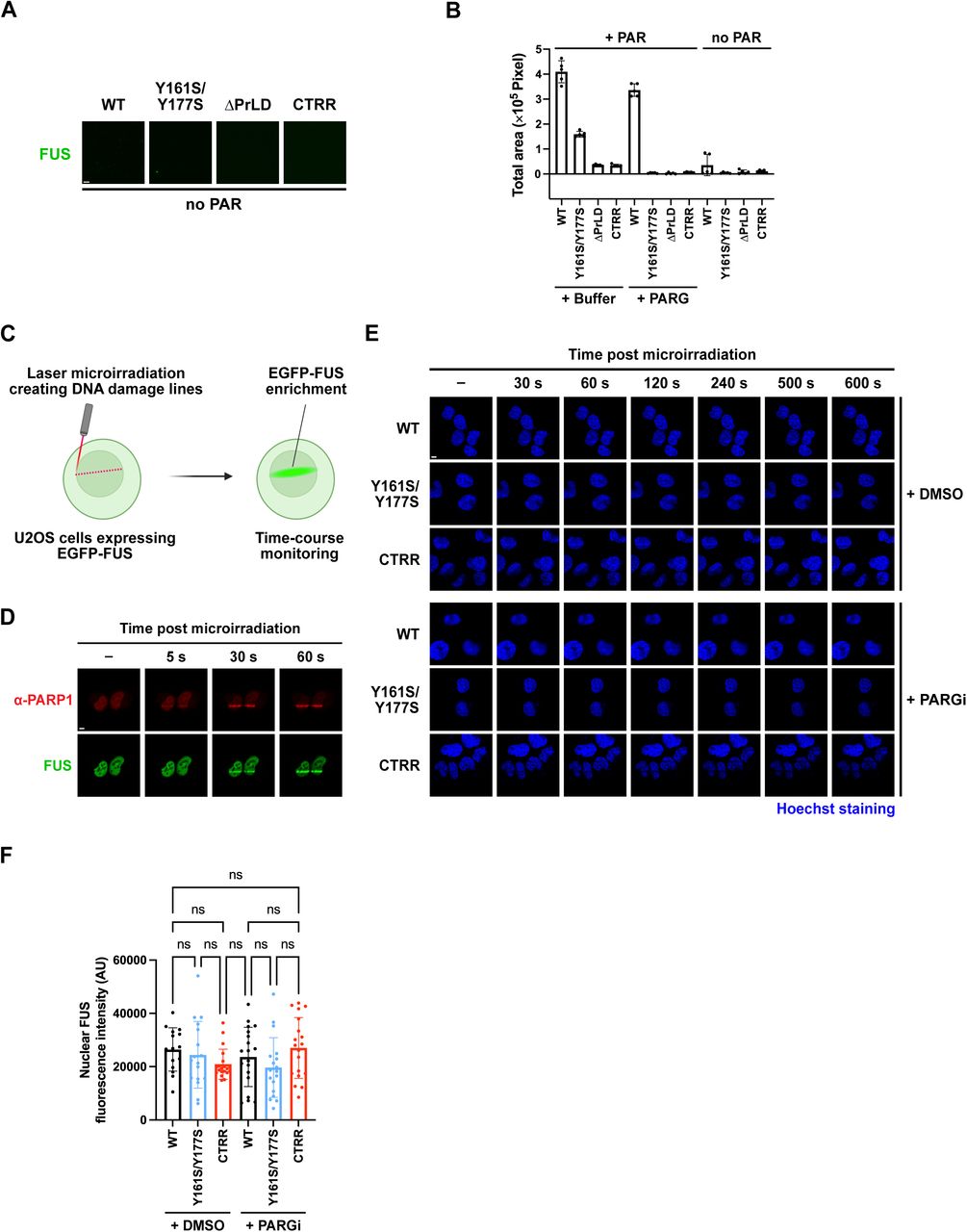

Supplement: Supplement 5 — Figure S5, related to Figure 5 (A) Representative microscopic images of condensates formed by 1 μM of wild-type FUS or corresponding mutants (green) without PAR after 2 h incubation. n = 5. Scale bar, 5 μm. See also (B). (B) Quantification of the total area covered by condensates formed in Figure 5J and S5A. n = 5; error bars, SD. (C) Schematic of the laser strip assay conducted in Figures 5K and 5M. Briefly, a 355 nm ultraviolet-A pulsed laser was used to microirradiate U2OS nuclei transiently transfected with EGFP-FUS, with time-course monitoring of FUS enrichment. (D) Representative time-course microscopic images of laser strip assays showing the early recruitment of endogenous PARP1 (red; detected using an RFP-tagged anti-PARP1 chromobody) and the subsequent enrichment of FUS within 1 min post-microirradiation. n = 5. Scale bar, 5 μm. (E) Hoechst staining indicating cell nuclei in the laser strip assays, corresponding to Figures 5K and 5M. Scale bar, 5 μm. (F) Bar graph showing the nuclear fluorescence intensity of FUS before microirradiation in wild-type FUS, the Y161S/Y177S mutant, and CTRR-expressing cells treated with either DMSO or PARG inhibitor. Each dot represents an individual cell. n ≥ 15; error bars, SD; ordinary one-way ANOVA with Tukey's multiple comparisons test, showing no significant differences (ns) between any conditions. [file figure-S5.jpg]
